# Endovascular Outcomes With One-Year Follow-Up of Three Cases of Spinal Arteriovenous Malformation (sAVM) at Cervical, Dorsal, and Lumbar Levels: A Single Institution-Based Case Series of a Rare Entity

**DOI:** 10.7759/cureus.78652

**Published:** 2025-02-06

**Authors:** Srinivas Gedala, Sreehari Nirmala Ramachandran, Malathi Mini, Dalvin Thomas, Sajesh K Menon

**Affiliations:** 1 Neurosurgery, Amrita Institute of Medical Sciences and Research Centre, Amrita Vishwa Vidyapeetham, Kochi, IND; 2 Public Health, Amrita Institute of Medical Sciences and Research Centre, Amrita Vishwa Vidyapeetham, Kochi, IND

**Keywords:** arteriovenous malformation, arteriovenous malformation (avm), endovascular embolisation, endovascular technique, spinal cord arteriovenous malformations, spinal cord myelopathy, spinal dural arterio-venous fistula

## Abstract

Spinal arteriovenous malformation (sAVM) is a rare vascular disease of the spine, presenting with symptoms of spinal cord involvement including sensory, motor, and autonomic symptoms. Early diagnosis is critical as the disease progresses to cause spinal cord compression, venous hypertension, and compression leading to spinal cord ischemia or infarction. In this case series, we present three cases treated at our institution over one year, with distinctly different anatomical sites of involvement. The three cases include two cases of type IV (perimedullary) AVMs - one at the C1-C2 level of the spinal cord and the other at the L1 level; and one case of type I (dural) AVM - at the D9-L1 level. Prompt diagnosis was made with the help of MRI and angiography. Endovascular embolization was performed for all patients, whose condition returned to normal within one year of follow-up.

## Introduction

Arteriovenous malformations (AVMs) are characterized by anomalous connections between arteries and veins without an intervening capillary network [1]. Spinal arteriovenous malformations (sAVMs) are rare but potentially devastating vascular anomalies of the spinal cord that can result in progressive neurological deterioration [2]. Although not completely understood, research suggests that spinal AVMs are congenital malformations that develop at three to eight weeks of gestation. The direct shunting of blood arteries and veins causes cellular injury due to the absence of pressure regulation by capillaries [1,3]. The epidemiology of sAVMs is sparsely described. However, the most common type is dural arteriovenous fistula [2]. Zozulya et al. have classified spinal AVMs as intramedullary, intradural or perimedullary, dural, epidural, intravertebral, and combined [4]. Major treatment modalities include surgical occlusion and endovascular embolization, with the latter emerging as a critical treatment modality for these lesions, even though the outcomes remain varied and dependent on multiple factors [3]. In this series, we discuss the cases of three patients with sAVM - at cervical, dorsal, and lumbar levels - who presented to our institution over one year. After prompt and early diagnosis, we proceeded with endovascular management, the outcomes of which differed slightly among the three.

## Case presentation

Case 1

Patient History

 A 27-year-old female presented with complaints of weakness of the left lower limb for three days and a history of neck pain with vomiting for one day. On admission, the patient was afebrile, with a blood pressure of 130/88 mmHg and a pulse rate of 88 bpm. On examination, motor powers in the left lower limb were 4/5 for hip flexion and extension 4/5 and 4/5 for knee and ankle joints. Motor powers in the bilateral upper limbs and right lower limb were 5/5. There was no history of any bowel and bladder disturbances and sensations were intact. Tone increased in the left lower limb with exaggerated deep tendon reflexes (DTRs). Plantar extensor was positive on the left side and mute on the right side. 

Diagnostic Workup

**Figure 1:**
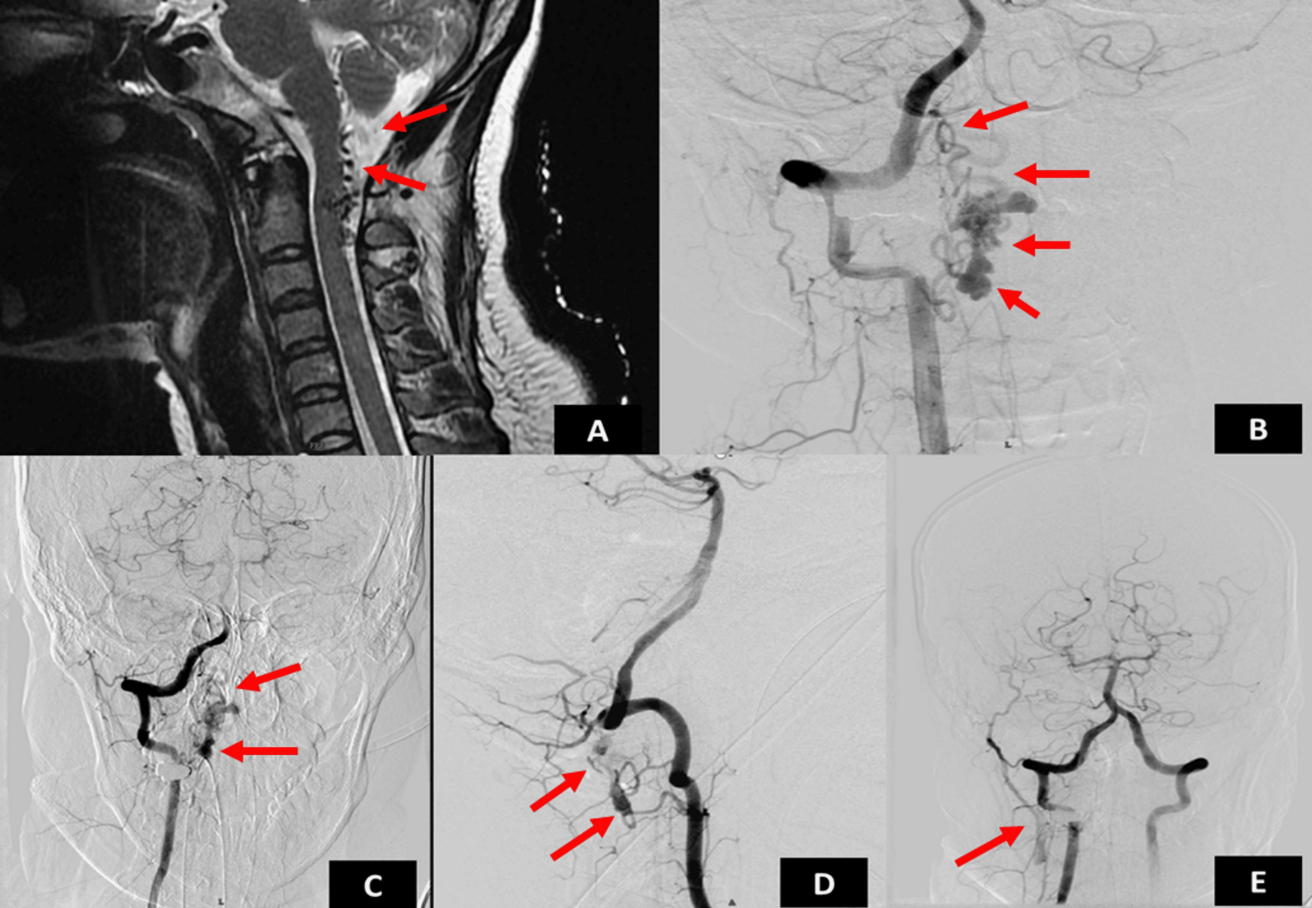
(A) Preoperative MRI of the patient. (B) DSA revealed AVMs in the intradural perimedullary (type IV- sub type II) C1-C2 segment of spinal cord. (C, D, E) Good occlusion of the AVM noted AVM: arteriovenous malformation; DSA: digital subtraction angiography; MRI: magnetic resonance imaging

**Figure 2:**
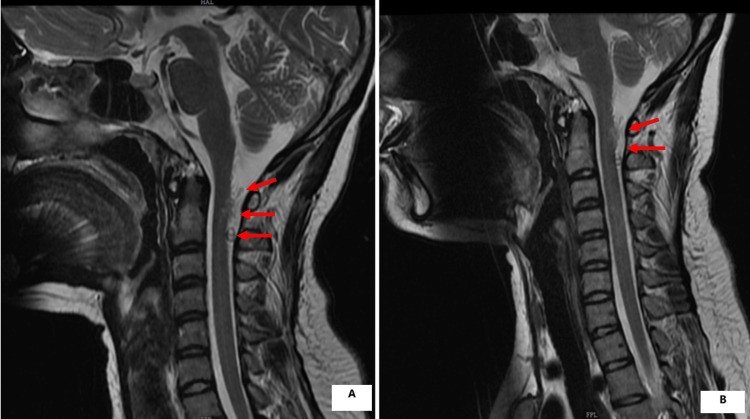
(A) Preoperative MRI of the patient. (B) Postoperative one-year follow-up MRI of the patient MRI: magnetic resonance imaging

MRI of the spine revealed flow voids in the posterior CSF space extending from the craniomedullary junction to the C2-C3 disc level with suspicion of extension into the spinal cord (Figures 1A, 2A). The gold standard investigation - digital subtraction angiography (DSA) - was done under local anesthesia, which revealed intradural perimedullary AVM (type IV, subtype II) with a tuft of the nidus of size 2 x 1.5 x 2 mm at C1-C2 segment of the spinal cord with feeders from the cervical branch of right vertebral artery and anterior spinal artery. The venous drainage was into ascending perimedullary veins with multiple venous ectasia, leading to a diagnosis of cervical AVM intradural perimedullary (type IV subtype II) - C1-C2 segment of the spinal cord (Figures 1B-1E).

Treatment

The patient underwent transarterial glue embolization through right femoral access, which resulted in an improvement of the motor power of the left hip. Post-treatment, the patient was followed up for three months. On follow-up, there was an improvement of motor power in the left lower limb - hip flexion and extension improved to 4+/5, knee and ankle joints improved to 3/5 and physiotherapy was continued. At the six-month follow-up, the motor power had improved to 5/5 on all four limbs. The patient was able to return to normal activities soon. At the one-year follow-up, there were no new deficits (Figure 2B).

Case 2

Patient History

A 51-year-old male presented with complaints of difficulty in walking due to weakness in the bilateral lower limbs, left more than the right, with tingling and numbness in extremities for four weeks. A history of severe low back pain was also present. The patient was a known case of chronic liver disease (CLD) Child-Pugh type 1 and diabetes mellitus. On admission, the patient was afebrile, with a blood pressure of 120/78 mmHg and a pulse rate of 82 bpm.

Neurological examination revealed motor power of grade 2/5 in the left hip and knee, and 4/5 in the right hip and knee. Motor power in the bilateral ankle was 3/5 and in the bilateral extensor hallucis longus (EHL) was 2/5. Tone increased in the four limbs. Sensations were reduced in bilateral lower limbs. DTRs were exaggerated bilaterally. Planters were mute bilaterally.

Diagnostic Workup

**Figure 3:**
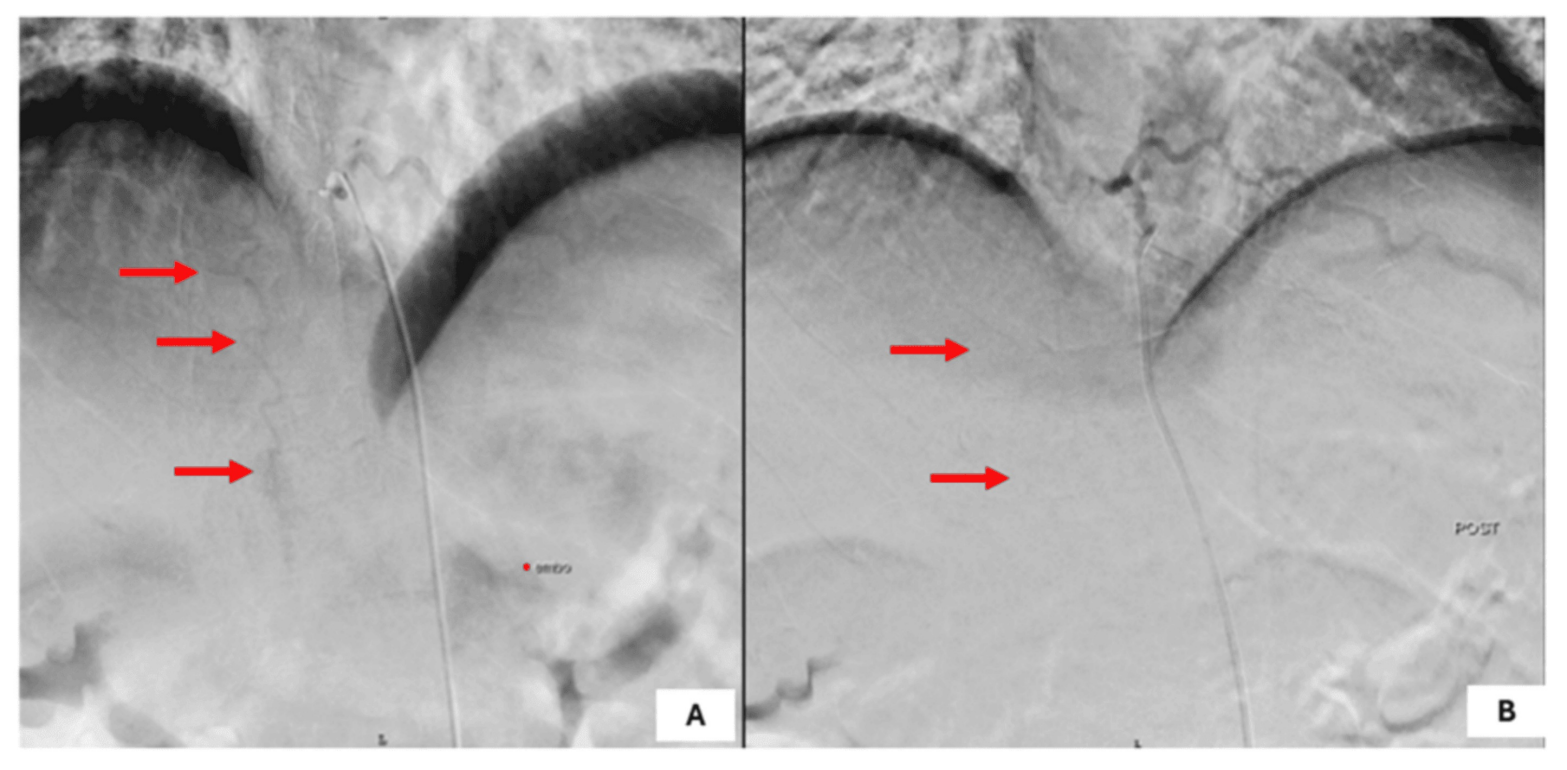
(A) Preoperative DSA image revealed AVMs at thoracolumbar spinal dural AVM D9-L1 with feeders from radiculomedullary branch arising from the lower intercostal artery. (B) Postoperative image showing good occlusion of AVM with no collaterals AVM: arteriovenous malformation; DSA: digital subtraction angiography

MRI of the spine revealed prominent flow voids in the spinal cord from D11 to L5 vertebral levels with probable feeder from the radiculomedullary branch arising from the lower intercostal artery suggestive of dural AVM (Figure 3A). At all these levels, spinal cord edema was also seen. DSA under local anesthesia revealed low-flow dorsal AVM with feeders from the D10 intercostal branch. A tuft of nidus was identified at the L1 level, with very small feeders seen from Intercostal branches. The intracranial vessels and iliac vessels were found to be normal (Figure 3B).

Treatment

The patient underwent transarterial glue embolization through right femoral access with a pigtail catheter. The D10 intercostal artery feeder was cannulated with an HH1 catheter. The microcatheter was navigated into the major feeder and embolized with 50/50% acrylic glue and lipoidol. After the embolization, nidus filling stopped. Other feeders were not accessible with microcatheters. However, during the procedure, after embolization, the patient developed severe back pain, due to which further catheter manipulation was not done. Follow-up DSA is planned for this patient to rule out any recruitment.

**Figure 4:**
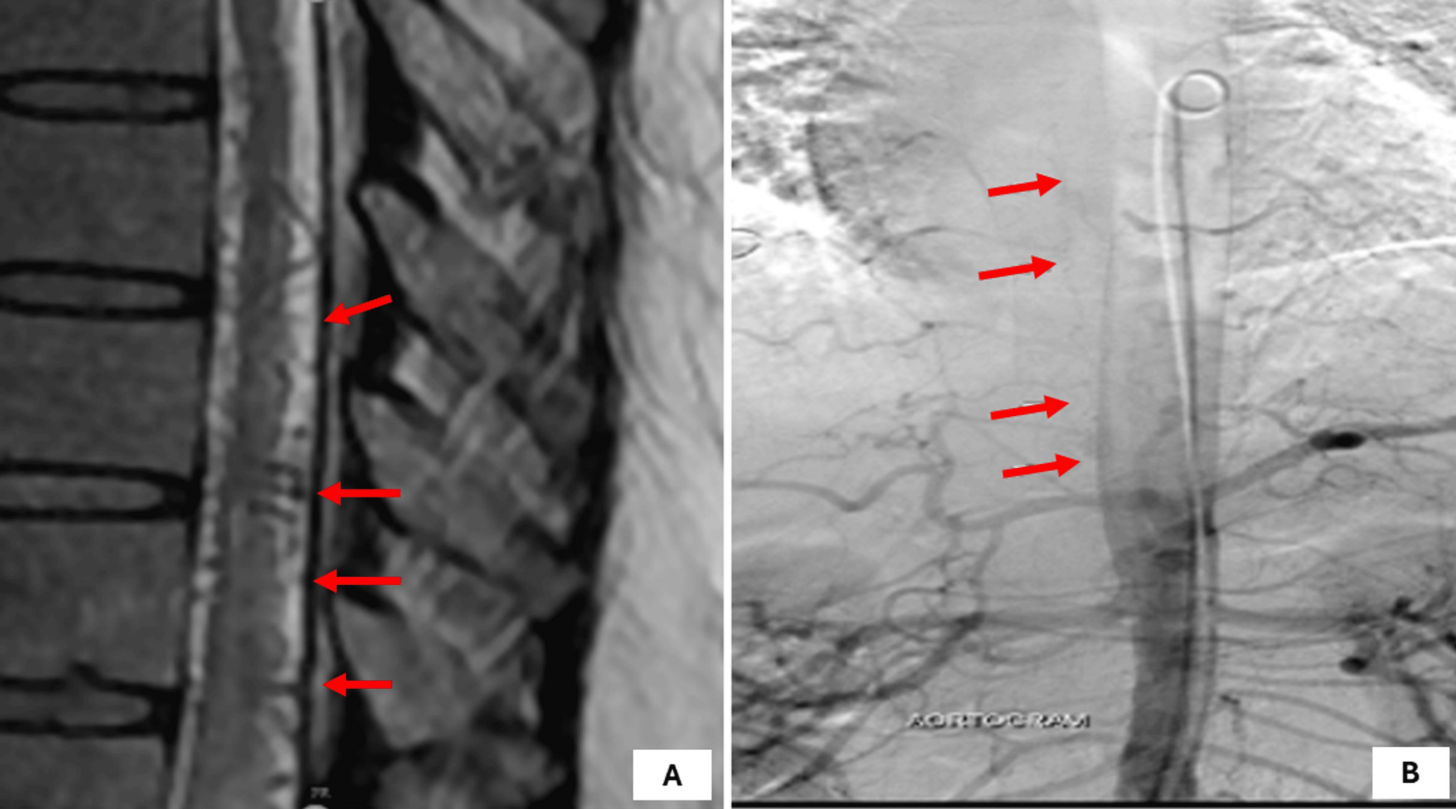
(A) Postoperative MRI and (B) DSA of the patient at one-year follow-up showing the recurrence of AVM AVM: arteriovenous malformation; DSA: digital subtraction angiography; MRI: magnetic resonance imaging

Post-treatment, the patient was followed up with MRI imaging after three months, which showed progression of the prior documented flow voids along the spinal cord, which were seen extending to the upper thoracic cord. Prior documented cord signal changes involving the distal spinal cord including the conus remained nearly the same. There were no features of cord atrophy. One-year follow-up MRI and DSA imaging showed minimal Dural AVM filling (Figure 4). The patient was posted for second-stage DSA and embolization. On follow-up, the patient's weakness had improved and he was able to perform his routine daily activities.

Case 3

Patient History

A 63-year-old male presented with complaints of difficulty in walking due to weakness of bilateral lower limbs. He also gave a history of buckling of the knees while walking. The patient was afebrile, with a blood pressure of 136/74 mmHg and a pulse rate of 82 bpm. Neurological examination revealed motor power of 4/5 for bilateral hip flexion and extension, 4/5 for knee flexion and extension, and 3/5 for ankle dorsi- and plantar flexion. Motor powers for EHL flexion and extension were 3/5. Sensations were reduced bilaterally from knees to feet. Bladder incontinence was also present.

Diagnostic Workup

**Figure 5:**
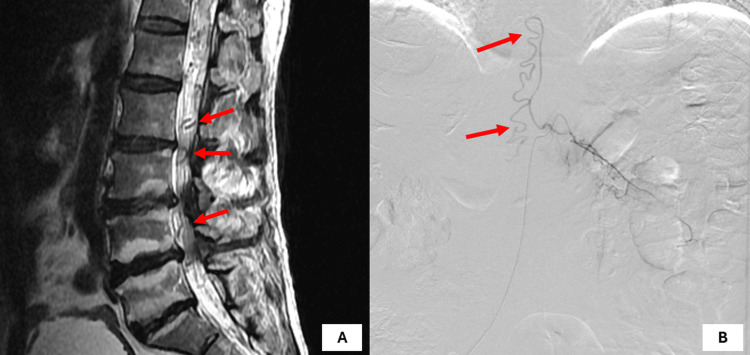
(A) Preoperative MRI and (B) DSA revealed AVM at L1 level (type 4A) AVM: arteriovenous malformation; DSA: digital subtraction angiography; MRI: magnetic resonance imaging

MRI of the spine revealed dural AVM with an arterial feeder from the radiculomedullary branch of the D12 intercostal artery on the left side. The dorsal cord showed diffuse T2 bright signals from D7 to conus, which were highly suggestive of cord edema with the concern of cord ischemia due to chronic dural AVM (Figure 5A). DSA was performed, which confirmed type 1 spinal dural AVM with arterial feeder from L1 lumbar radicular artery with delayed venous drainage into the perimedullary venous plexus with the presence of nidus at L4 level. Evidence of tortuous arterial feeder was also identified (Figure 5B).

Treatment

**Figure 6:**
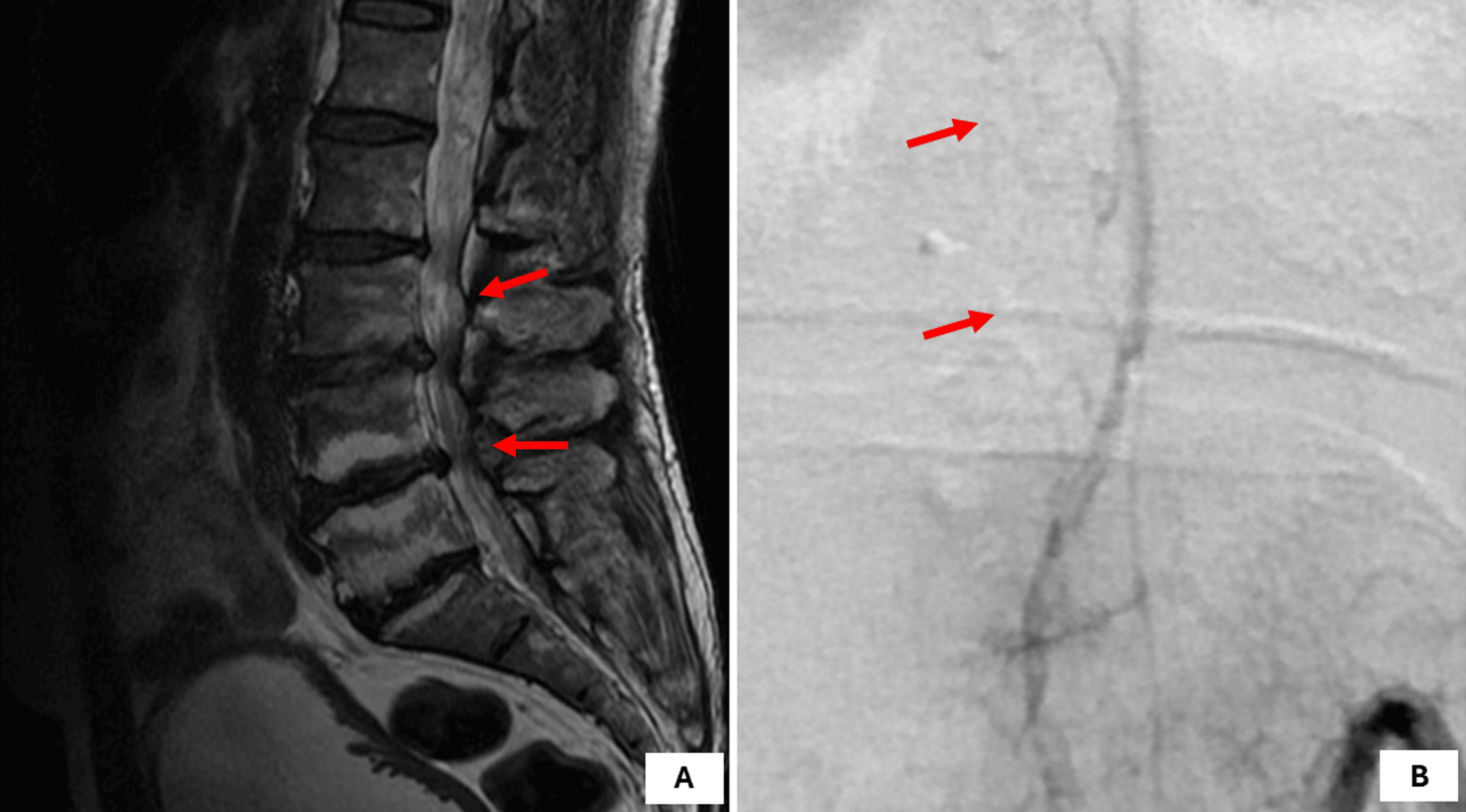
Postoperative (A) MRI and (B) DSA of the patient at the six-month follow-up showing no recruitment DSA: digital subtraction angiography; MRI: magnetic resonance imaging

The patient underwent trans arterial glue embolization through right femoral access, which revealed a low flow, type 1 spinal dural AVM with arterial feeder from L1 lumbar radicular artery with delayed venous drainage into the perimedullary venous plexus. Under all aseptic precautions, using the transfemoral route and modified Seldinger technique, a 6F arterial sheath was inserted and the L1 lumbar artery was cannulated using Cobra 5F. Using Magic 1.5F, the arterial feeder cannulated distally with the help of a microwire. Glue embolization was done using NBCA:Lipiodol in a 30:70 ratio. Post-procedure injection showed no filling of the AV malformation, with glue cast in the arterial feeder. Following this, the catheter was removed with the sheath kept in situ. Post-treatment, at the three-month follow-up, bilateral lower limb power improved to 4/5 in hips, 4/5 in knee extension, 3/5 in knee flexion, 3/5 in ankle, and 3/5 in EHL with bilateral mute plantar reflexes. However, urinary incontinence persisted and the patient was catheterized. At the one-year follow-up, bilateral lower limb power further improved, albeit with persisting incontinence (Figure 6).

## Discussion

We presented three cases of sAVMs treated at our institution within one year. To the best of our knowledge, this is the first report that describes cases with three different anatomical sites of occurrence in three patients (cervical, dorsal, and lumbar spine). While all of them were successfully managed by endovascular embolization, for one patient (case 2), further embolization needs to be done. This report highlights the advantage of diagnosing sAVM critically early and managing it without surgery to increase the chances of recovery. All of our patients had resumed normal activities in life by one-year follow-up.

The entity of sAVM includes both typical AVM characterized by a nidus of vessels between arteries and veins, and clinically present with sudden onset pain or weakness along with myelopathy or radiculopathy [3,5]. Both types of sAVMs together account for about 3-4% of all spinal cord mass lesions that are intradural. While extensive epidemiological studies about the condition are lacking, one study has reported a slightly higher prevalence among males [5,6]. The major differential diagnoses considered during clinical presentations include spinal epidural hematoma, spinal cord tumors, multiple sclerosis, syringomyelia, traumatic spinal injury, and hereditary hemorrhagic telangiectasia [7]. These conditions affect the spinal cord and present with motor and sensory deficits. However, in our case, the absence of any constitutional symptoms and radiological findings helped to clinch the diagnosis.

The Anson and Spetlzer classification of sAVM (1992) describes four variants: spinal dural arteriovenous fistula (type I), intramedullary AVM (type II), extradural-intradural AVM (type III), and intradural perimedullary arteriovenous fistula (type IV) [5]. Among these, type I is the most common and affects men more than women, and it is often found in the thoracic region [8]. Intramedullary (type II) is associated with higher mortality rates, while intradural perimedullary (type IV) is relatively rare with three subtypes depending on the type and flow in the feeder vessels [3,8]. Type IV usually affects lower thoracic or lumbar regions [2,3]. These malformations can have profound clinical consequences due to their potential to cause hemorrhage, ischemia, or mass effect on neural tissues by altering the hemodynamics [7].

The clinical presentations of the three cases in the report are typical of the anatomical locations of involvement. Case 1 (cervical) presented with neck pain, limb weakness, and signs of myelopathy; while case 2 (dorsal) presented with limb weakness and radiculopathy; and case 3 (lumbar) presented with limb weakness, bladder disturbances, and signs of myelopathy. Cervical AVMs usually present with myelopathy, progressive weakness in all limbs, sensory disturbances, and bowel and bladder disturbances, while thoracic and lumbar AVMs typically present with mid- and lower back pain respectively, along with sensory disturbances and autonomic dysfunction [3,5]. Progressive myelopathy is a common feature across all levels, typically beginning with sensory disturbances followed by motor deficits and sphincter dysfunction [3,5,7]. Due to the nonspecific nature of the early symptoms, diagnosis could be challenging. However, as elaborated above, timely and appropriate investigations can help save the patient from further deterioration. MRI is the first imaging modality, while DSA is the gold standard investigation [8].

Treatment options include microsurgical resection for accessible AVMs; endovascular embolization, which is the most preferred treatment option, especially for type I and type IV AVMs; and stereotactic radiotherapy [8,9]. A systematic review investigating the recurrence rates following various modalities for sAVMS reported initial treatment success of 70.6% and 60.5% following endovascular embolization for type I and type IV sAVMs respectively [10]. For type IV AVMs, the overall obliteration rate was 90% for type A lesions, 79% for type B, and 71% for type C lesions [11].

## Conclusions

This case series underscores the importance of considering sAVM in the differential diagnosis of patients presenting with myelopathy. Early identification and appropriate treatment are crucial for achieving positive outcomes in these rare presentations. Awareness of the sAVM is essential for radiographic screening of spinal lesions with myelopathy. The clinical therapeutic strategy should be multidisciplinary and individualized based on vasculature and lesion behavior. The rapid pace of innovations in the realm of endovascular techniques and devices continues to move the field toward broader indications and improved patient outcomes. Although much of the treatment success for sAVMs remains dependent on the type of lesion, endovascular therapies are becoming increasingly effective as both primary and adjunct therapies.
